# Case Report: Interleukin-17A blockade for SAPHO syndrome presenting as an FDG-avid destructive osteolytic acetabular lesion

**DOI:** 10.3389/fimmu.2026.1988760

**Published:** 2026-10-23

**Authors:** Shu Liu, Anning Wang, Yiming Yuan, Xiaomin Yan, Xianfeng Yang, Qiong Jiao, Zhiyong Chen, Shiying Wang

**Affiliations:** 1 Department of Rheumatology and Immunology, Nanjing Drum Tower Hospital, Affiliated Hospital of Medical School, Nanjing University, Nanjing, China; 2 Department of Infectious Diseases, Nanjing Drum Tower Hospital, Affiliated Hospital of Medical School, Nanjing University, Nanjing, China; 3 Department of Radiology, Nanjing Drum Tower Hospital, Affiliated Hospital of Medical School, Nanjing University, Nanjing, China; 4 Department of Pathology, Shanghai Sixth People’s Hospital Affiliated to Shanghai Jiao Tong University School of Medicine, Shanghai, China; 5 Department of Rheumatology and Immunology, Shanghai Sixth People’s Hospital Affiliated to Shanghai Jiao Tong University School of Medicine, Shanghai, China

**Keywords:** autoinflammation, case report, interleukin-17A, osteolytic lesion, paradoxical pustulosis, SAPHO syndrome, secukinumab, sterile osteitis

## Abstract

SAPHO syndrome is an autoinflammatory osteitis disorder that may be misdiagnosed when bone destruction dominates the presentation. We report a 27-year-old woman with 7 months of left hip pain and intermittent anterior chest-wall pain. Imaging revealed an FDG-avid destructive osteolytic lesion in the left acetabulum and adjacent ilium, initially raising concern for malignancy, lymphoma, Langerhans-cell histiocytosis, or infection. Repeated tissue sampling showed chronic sterile osteomyelitis-like inflammation without evidence of malignancy or a reproducible pathogen. The disease evolved with pustular eruption after tumor necrosis factor inhibition, insufficient axial imaging control with Janus kinase inhibition, and marked improvement of a T12 vertebral-corner inflammatory lesion after interleukin-17A blockade with secukinumab. This case highlights the value of longitudinal clinicopathologic reassessment in destructive inflammatory bone disease and suggests that IL-17A blockade may be a rational strategy for SAPHO syndrome with active axial osteitis and pustular inflammation.

## Introduction

SAPHO syndrome, encompassing synovitis, acne, pustulosis, hyperostosis, and osteitis, is a rare disorder that bridges autoinflammatory bone disease, neutrophil-rich skin inflammation, and spondyloarthritis-spectrum disease (1–4). Classic presentations involve the anterior chest wall, spine, sacroiliac joints, or long bones, often with hyperostosis or osteitis. However, the phenotype is heterogeneous, and skin manifestations can be absent at disease onset or appear later during the disease course. This variability makes early recognition difficult, particularly when a solitary destructive bone lesion is the dominant abnormality.

An FDG-avid osteolytic lesion in a young adult appropriately triggers evaluation for malignancy, histiocytic disorders, plasma-cell neoplasms, lymphoma, and infection. Yet sterile osteitis in SAPHO syndrome and chronic recurrent multifocal osteomyelitis can also produce marrow edema, cortical destruction, inflammatory histology, and increased metabolic activity. The clinical challenge is not simply naming a rare diagnosis, but repeatedly recalibrating the probability of tumor, infection, and autoinflammatory disease as pathology, microbiology, imaging, treatment response, and new organ manifestations accumulate. SAPHO syndrome is now better understood as an autoinflammatory disorder of the IL-23/IL-17 axis than as a simple infection of bone, and this shift in conceptual framing has practical consequences for diagnosis, for the interpretation of biopsy and molecular microbiology results, and for treatment selection (4, 17, 19). We report a case in which this longitudinal process ultimately supported SAPHO syndrome and guided escalation to IL-17A blockade, and we compare the presentation with previously reported cases of tumor-mimicking osteolytic SAPHO syndrome and review the emerging treatment landscape.

## Case description

A 27-year-old woman was evaluated in the rheumatology clinic because of a 7-month history of left hip pain and intermittent pain in the left upper anterior chest wall near the costochondral region. She had no fever, weight loss, uveitis, inflammatory bowel symptoms, oral-genital ulceration at presentation, or small-joint arthritis. Initial treatment at a community hospital for presumed costochondritis, including nonsteroidal anti-inflammatory and topical analgesic therapy, did not provide meaningful relief.

Two months after symptom onset, hip pain worsened. MRI and computed tomography performed elsewhere showed marrow edema in the left acetabulum and adjacent ilium, edema in surrounding soft tissue, and an osteolytic destructive lesion involving cortical bone. PET-CT showed increased FDG uptake at the left acetabular lesion. The initial radiologic differential diagnosis included aggressive tumor, lymphoma, Langerhans-cell histiocytosis, and infection (**Figure 1**).

**Figure 1:**
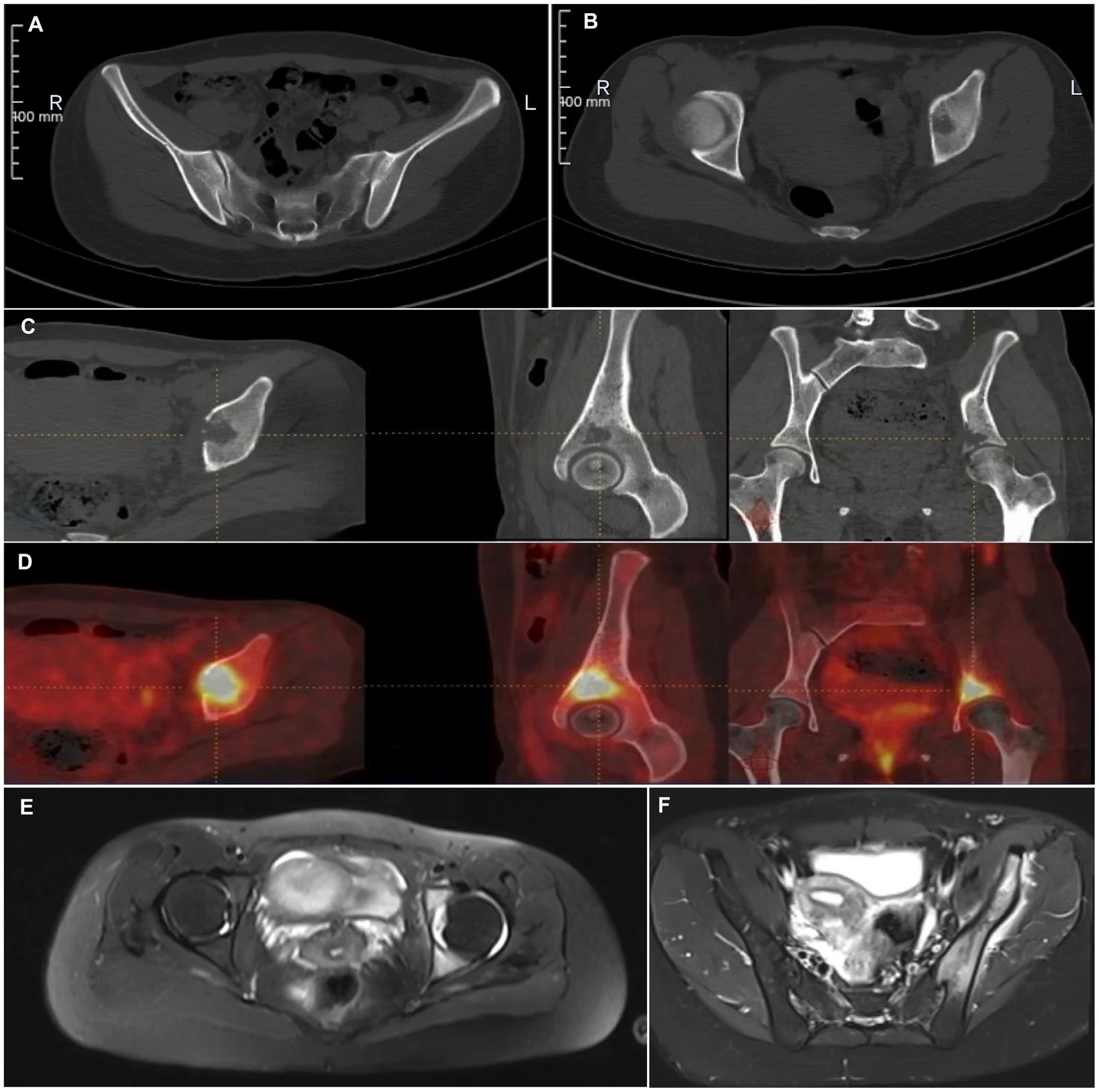
Initial imaging of the pelvis and left acetabulum. Axial computed tomography showed a destructive osteolytic lesion in the left acetabulum and adjacent ilium **(A–C)**. FDG PET-CT showed intense uptake at the lesion **(D)**. Pelvic MRI showed marrow edema and adjacent soft-tissue edema **(E, F)**.

A computed tomography-guided core biopsy showed fibrogranulation tissue and an epithelioid granulomatous inflammatory process with abundant acute and chronic inflammatory-cell infiltration. Immunohistochemical staining was negative for epithelial markers, CD1a, S100, Langerin, ALK, and OCT2, and positive for CD163. Metagenomic next-generation sequencing (mNGS) of paraffin-embedded tissue detected low-confidence Nocardia farcinica; however, the same organism was detected in the water bath used for slide preparation, and peripheral-blood microbial cell-free DNA testing was negative. At that outside-hospital stage, the working diagnosis remained an aggressive osteolytic tumor or tumor-like lesion, with Langerhans-cell histiocytosis or lymphoma still under consideration and no confirmed infection or SAPHO diagnosis. Denosumab, 120 mg subcutaneously every 4 weeks, was therefore administered as antiresorptive treatment for presumed tumor-like osteolytic bone destruction, not as disease-specific therapy for SAPHO syndrome. The treatment was associated with transient symptomatic improvement. After approximately 4 months of denosumab therapy, hip pain worsened again and MRI showed progression of abnormal signal in the left iliac wing, acetabulum, superior pubic ramus, and surrounding soft tissues (**Supplementary Figure 1A**).

At the index rheumatology evaluation, multidisciplinary review considered infection, tumor, histiocytic disease, and sterile autoinflammatory osteitis. Given the osteolytic appearance, inflammatory pathology, and uncertain mNGS result, empirical antimicrobial therapy with intravenous imipenem-cilastatin and linezolid was started. After discharge, oral linezolid and minocycline were continued for 14 days. The left hip symptoms did not substantially improve. One month after discharge, partial resection, curettage, bone grafting, and internal fixation of the left acetabular lesion were performed. The operative specimen showed marrow fibrosis with dense chronic inflammatory-cell infiltration, predominantly T lymphocytes, and CD68-positive histiocytes; CD1a, CD20, and Langerin were negative, and tissue mNGS detected no pathogen (**Figure 2**). These findings shifted the working diagnosis away from neoplasm or proven infection and toward sterile chronic osteitis. The chronological evolution supporting SAPHO syndrome was therefore cumulative: an FDG-avid destructive sterile osteitis-like lesion, anterior chest-wall pain, repeat negative tissue testing for tumor and reproducible pathogens, later pustular disease with granulomatous folliculitis, and subsequent sacroiliitis and vertebral-corner inflammatory lesions. The episode-of-care timeline is detailed in **Table 1**.

**Figure 2:**
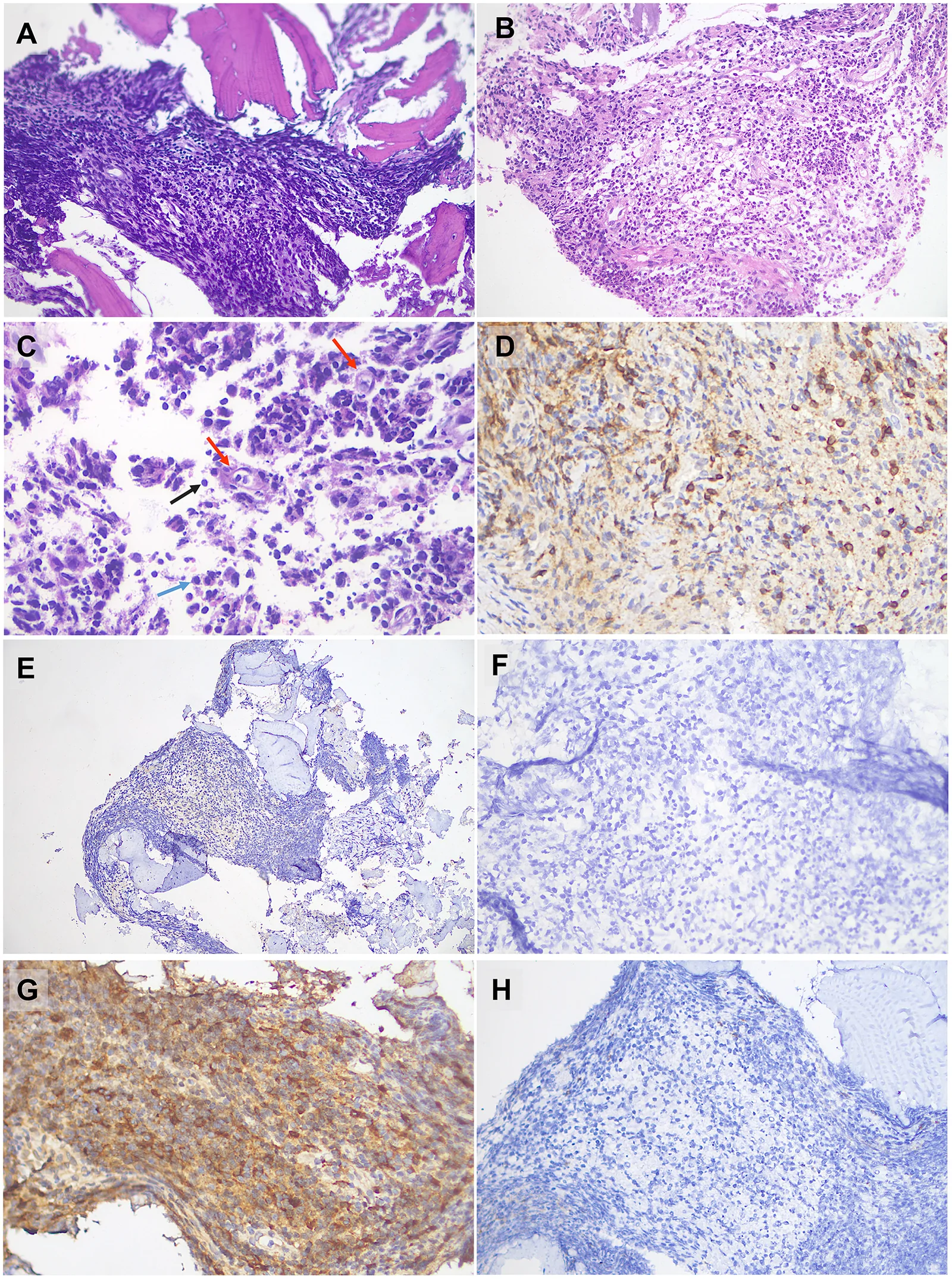
Pathological findings in the acetabular bone-biopsy and operative specimens. Hematoxylin and eosin staining showed fibrogranulation tissue, chronic inflammatory-cell infiltration, and vascular proliferation around bone fragments **(A-C)**. In the high-power image **(C)**, arrows indicate proliferating small vessels (red), lymphocytes (black), and plasma cells (blue) within the mixed inflammatory infiltrate. Immunohistochemical staining showed scattered CD3-positive T lymphocytes **(D)**, no epithelial component on cytokeratin staining **(E)**, negative EBER *in situ* hybridization **(F)**, abundant CD68-positive histiocytes **(G)**, and no prominent CD20-positive B-cell infiltrate **(H)**.

**Table 1:** Timeline of the episode of care.

Time point	Clinical/imaging findings	Intervention or interpretation
7 months before evaluation	Left hip pain and intermittent left anterior chest-wall pain began.	Community diagnosis of costochondritis; nonsteroidal anti-inflammatory and topical analgesic treatment did not provide meaningful improvement.
5 months before evaluation	MRI/CT showed left acetabular and iliac marrow edema, surrounding soft-tissue edema, and osteolytic bone destruction.	PET-CT showed increased FDG uptake; tumor, lymphoma, Langerhans-cell histiocytosis, and infection were considered.
After initial biopsy	Core biopsy showed epithelioid granulomatous inflammation with mixed acute and chronic inflammatory-cell infiltration; CD163 positive; CD1a, S100, Langerin, ALK, OCT2, and epithelial markers negative.	Low-confidence Nocardia farcinica was detected in paraffin tissue mNGS, but water-bath contamination and negative peripheral-blood microbial cfDNA weakened causality. Denosumab 120 mg every 4 weeks was given.
Index rheumatology evaluation	Pain recurred with abnormal pelvic MRI; MDT reviewed infection, tumor, histiocytic disease, and sterile osteitis.	Empirical imipenem-cilastatin and linezolid followed by oral linezolid/minocycline did not substantially improve hip symptoms.
1 month after discharge	Partial resection, curettage, bone grafting, and internal fixation were performed.	Operative pathology showed chronic osteomyelitis-like inflammation dominated by T lymphocytes and CD68-positive histiocytes; tissue mNGS detected no pathogen. SAPHO syndrome was diagnosed.
After adalimumab initiation	Adalimumab 40 mg every 2 weeks improved joint symptoms initially, but pustular lesions appeared after the first injection and progressively worsened.	Skin biopsy supported granulomatous folliculitis in the context of SAPHO syndrome; adalimumab was stopped.
Subsequent treatment	Apremilast and cyclosporine were ineffective. Upadacitinib 15 mg daily improved the eruption, but pelvic/spine MRI progressed with bilateral sacroiliitis and vertebral-corner lesions, especially T12.	Persistent axial osteitis prompted escalation.
After secukinumab	Secukinumab 150 mg weekly for 5 doses, then every 2 weeks, was started.	Approximately 2 months later, T12 MRI inflammation improved markedly, pustular disease did not recur, gait improved, and maintenance dosing was extended to every 3 weeks.

## Diagnostic assessment

The final diagnosis was SAPHO syndrome according to the modified Kahn diagnostic criteria, after exclusion of infection, malignancy, and histiocytic disease (2). The patient fulfilled the criteria through sterile chronic osteitis or osteomyelitis-like bone inflammation, anterior chest-wall involvement, later pustular skin disease, and subsequent axial inflammatory lesions. The diagnosis was not apparent at onset because the dominant presentation was an FDG-avid destructive acetabular lesion rather than the more familiar sternoclavicular hyperostosis.

Several alternative diagnoses were considered and sequentially downgraded. Carcinoma and metastatic disease were unlikely because repeated pathology did not show cohesive epithelial malignant cells, epithelial markers were negative, and systemic imaging did not reveal a primary tumor. Lymphoma was not supported by the pathology, immunophenotype, or lack of a prominent clonal B-cell infiltrate. Langerhans-cell histiocytosis was disfavored by negative CD1a, S100, and Langerin staining. Other histiocytic disorders, including Erdheim-Chester disease and Rosai-Dorfman-Destombes disease, were considered because CD68-positive histiocytes were present, but the distribution, morphology, immunophenotype, and subsequent spondyloarthritis-like evolution were not typical (7–9).

Infection remained the most important competing diagnosis early in the course. Osteolytic destruction, soft-tissue edema, chronic inflammatory histology, and granulomatous features can occur in bacterial, mycobacterial, or fungal osteomyelitis. However, the evidence for Nocardia infection was not reproducible and was weakened by possible water-bath contamination, negative peripheral-blood microbial cfDNA testing, negative repeat tissue mNGS, absence of a sustained systemic infectious phenotype, and poor response to antimicrobial therapy. Thus, the pathology was interpreted as chronic osteomyelitis-like inflammation rather than proof of active bacterial infection (6).

This distinction was clinically consequential. If the lesion had been interpreted as proven infection, prolonged antimicrobial therapy or repeated debridement could have delayed control of the systemic inflammatory process. Conversely, premature immunosuppression before adequate tissue sampling would have been unsafe because the imaging appearance was destructive and metabolically active. The final diagnosis therefore depended on the cumulative direction of evidence rather than any single feature: the mNGS result became less persuasive after contamination was identified, whereas the sterile inflammatory pattern became more persuasive as chest-wall pain, pustular disease, sacroiliitis, and vertebral lesions emerged.

## Therapeutic intervention, follow-up, and outcomes

After infection and malignancy became less likely, the diagnosis was reframed as SAPHO syndrome, and treatment was redirected toward inflammatory osteitis. Adalimumab, 40 mg subcutaneously every 2 weeks, was started to control osteoarticular inflammation. Joint symptoms improved after the first two injections, suggesting that TNF-related inflammation was active. However, pustular lesions appeared after the first injection and became progressively extensive with continued treatment, involving the scalp, face, trunk, and limbs (**Figures 3A–D**). Skin biopsy showed epidermal hyperplasia, acanthosis, perifollicular lymphocytic inflammation, follicular dilatation, and histiocytic and multinucleated giant-cell deposition, consistent with granulomatous folliculitis in the context of SAPHO syndrome (**Supplementary Figure 2**). This cutaneous phase developed after the initial bone presentation and further supported the evolving SAPHO-spectrum diagnosis. Adalimumab was discontinued. After treatment with doxycycline, methylprednisolone, and topical fusidic acid, repeat MRI showed partial improvement 3 months after discharge (**Supplementary Figure 1B**).

**Figure 3:**
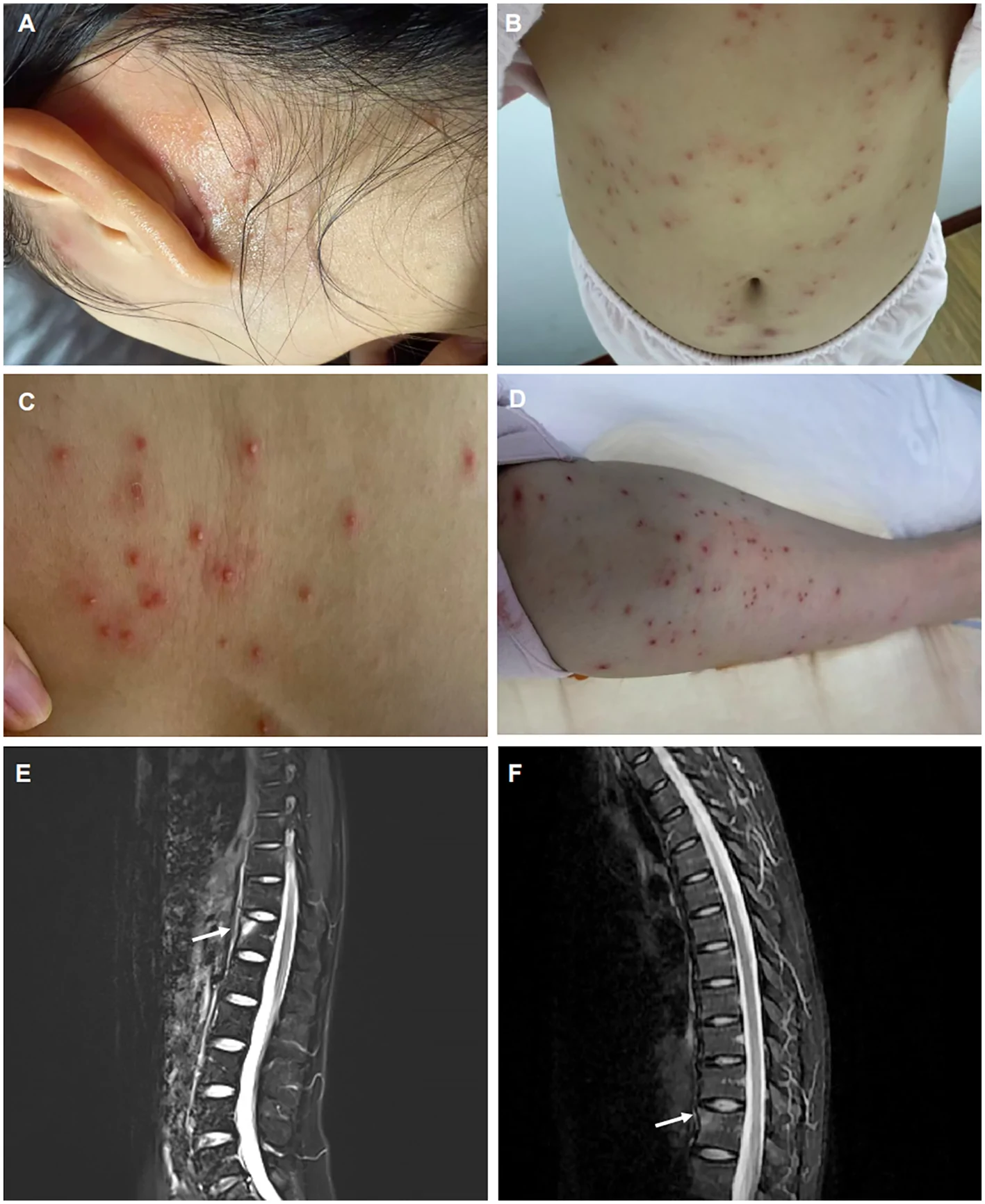
Pustular dermatitis and spinal MRI response during immunomodulatory therapy. Pustular and erythematous lesions progressively involved the scalp, trunk, and limbs after initiation of adalimumab **(A–D)**. Sagittal fat-suppressed T2-weighted MRI showed an inflammatory lesion at the T12 vertebral-body corner before secukinumab treatment **(E)**, arrow). Approximately 2 months after secukinumab was started, repeat MRI showed marked improvement of the T12 lesion **(F)**, arrow).

Subsequent treatment with apremilast and cyclosporine was ineffective. During glucocorticoid tapering, MRI showed recurrent progression of marrow and soft-tissue edema (**Supplementary Figure 1C**). The axial inflammatory phenotype became clearer later in the course: upadacitinib, 15 mg daily, improved the eruption clinically, but follow-up pelvic MRI showed progression to bilateral sacroiliitis, more prominent on the left, with irregular articular surfaces, erosions, marrow edema, and fat deposition (**Supplementary Figure 1D**). Spine MRI showed inflammatory lesions at multiple vertebral-body corners, especially T12 (**Supplementary Figure 1E**). Because active axial osteitis persisted despite clinical skin improvement, secukinumab was administered at 150 mg weekly for five doses, followed by 150 mg every 2 weeks. Approximately 2 months after initiation, repeat MRI showed marked improvement of the T12 vertebral-corner inflammatory lesion; the pustular eruption had not recurred, gait had improved, and the maintenance interval was later extended to every 3 weeks (**Figures 3E, F**; **Supplementary Figure 1E**).

The treatment goal was therefore broadened from analgesia or suppression of skin inflammation to control of objective axial osteitis. This was important because the patient reported symptomatic fluctuations, whereas MRI showed that inflammatory activity persisted despite partial clinical improvement. Serial imaging was used to distinguish active inflammatory lesions from residual structural damage in the acetabulum and sacroiliac joints. The absence of recurrent pustular disease during secukinumab maintenance, together with improved gait and reduced vertebral-corner edema, suggested a more integrated response across skin and bone domains.

## Discussion

This case is distinctive because SAPHO syndrome initially presented as a metabolically active destructive osteolytic acetabular lesion, a pattern that strongly mimicked tumor or chronic infection. The major strength of the diagnostic approach was repeated tissue sampling and longitudinal reassessment rather than reliance on a single test result. The initial biopsy raised infection as a plausible explanation, but the microbiologic signal was discordant and potentially contaminated. The operative specimen provided a second opportunity to reassess the lesion and showed chronic sterile osteomyelitis-like inflammation without a reproducible pathogen or malignancy.

Several features of this presentation have been described previously. Kinoshita et al. described a 54-year-old woman with palmoplantar pustulosis in whom a purely osteolytic femoral lesion with intense FDG uptake on PET-CT was attributed to bone metastasis or chronic bacterial osteomyelitis, with the diagnosis established only after needle biopsy showed no malignancy and cultures were negative; notably, the lesion in that case converted from purely osteolytic to osteosclerotic over 15 months, a transition not documented in the present case (14). Yang et al. reported three patients in whom SAPHO syndrome was initially managed as spinal infection at outside institutions, in whom PET-CT again demonstrated FDG-avid osteolytic or mixed lesions and in whom diagnosis required systematic exclusion of infection and neoplasm (15). The 164-patient single-center cohort provides useful denominators for comparison: anterior chest-wall pain was present in all patients, only 2.4% were HLA-B27 positive, and skin involvement was absent in a small but non-negligible minority, confirming that a bone-first presentation without skin disease is a recognized, if uncommon, SAPHO phenotype rather than an anomaly (16). Our case shares the tumor-mimicking radiologic phenotype and the delayed, diagnosis-driven treatment sequence described in these reports, but differs in three respects. First, the destructive lesion was centered on the acetabulum and adjacent ilium, an axial appendicular site that is not the classic anterior chest-wall or sternoclavicular location emphasized in the diagnostic criteria, and the correct diagnosis was therefore reached only after the anterior chest-wall component of the history was integrated (2, 16). Second, the microbiologic evaluation was more granular than in previous reports: a low-confidence Nocardia farcinica signal by tissue metagenomic sequencing was subsequently shown to be non-reproducible and attributable to water-bath contamination, which is a specific pitfall that deserves wider recognition because a single positive molecular result in this setting can wrongly anchor the diagnosis to infection (6). Third, and most importantly, this case is distinguished by the sequential emergence of a pustular and axial inflammatory phenotype during treatment and by the observation that skin and bone domains responded discordantly, with skin improvement under JAK inhibition accompanied by radiographic progression, followed by improvement of an objective vertebral-corner lesion after IL-17A blockade. Prior reports of secukinumab in SAPHO syndrome have chiefly emphasized cutaneous responses, including paradoxical psoriasis after adalimumab (21) and prevention of recurrent musculoskeletal pain in a patient with coexisting ankylosing spondylitis (22); systematic and narrative syntheses likewise conclude that IL-17A evidence is limited to case reports and small series (5, 19). The distinguishing feature of the present case is therefore not the use of secukinumab itself, but the use of serial MRI as the primary efficacy endpoint, which converts a subjective treatment response into an objective demonstration of suppressed axial osteitis.

The mechanisms underlying SAPHO syndrome remain incompletely resolved, but a coherent framework has emerged from genetic, immunological, and microbiological observations. The syndrome is generally classified as autoinflammatory rather than classically autoimmune, based on neutrophil hyperactivity, reduced numbers of circulating natural killer cells, elevated levels of IL-1, IL-6, IL-8, IL-17, IL-18, and TNF, and clinical responses to IL-1 blockade (4, 17, 19). At the center of this model sits the T helper 17 (Th17)-IL-23-IL-17 axis. IL-23 sustains IL-17A production, and IL-17A in turn drives both the neutrophil-rich cutaneous inflammation of palmoplantar pustulosis and the osteoarticular manifestations, in the latter case by promoting osteoclast differentiation and activation through receptor activator of nuclear factor kappa-B ligand (RANKL)-dependent pathways. This dual action explains why SAPHO syndrome produces the seemingly paradoxical combination of osteolysis in early active disease and hyperostosis in later stages, and it is also why skin and bone involvement can dissociate, as they did in this patient under JAK inhibition (5, 11, 19). Genetic studies have not identified a monogenic cause. Candidate loci including LPIN2, PSTPIP2, NOD2, and FBLIM1 have been proposed but not confirmed across cohorts, and a genome-wide association study identified aberrant osteoclast differentiation and related inflammatory pathways, supported by transcriptome evidence of overactive neutrophil recruitment genes, as the most consistently implicated signal rather than any single Mendelian variant (4, 19, 20). A unifying structural concept is that SAPHO syndrome, chronic nonbacterial osteitis, and chronic recurrent multifocal osteomyelitis represent overlapping points on a single autoinflammatory spectrum of sterile osteitis, differing mainly in age of onset, distribution of bone involvement, and the prominence of associated skin disease; this framework also places SAPHO syndrome within the broader PAPA and PASH/PSTOP group of autoinflammatory conditions, which share sterile osteitis, neutrophilic dermatosis, and psoriatic or acneiform skin disease (4, 18, 19). An infectious or microbial contribution has long been hypothesized but remains unproven. Cutibacterium acnes and, more recently, gut-derived Prevotella species have been implicated, the latter capable of inducing IL-1beta and IL-23 and promoting a Th17 response in experimental models, and humoral abnormalities including elevated IgG4 and monoclonal immunoglobulin have also been described. None of these observations establish a single causative organism, and the modest response of SAPHO syndrome to antibiotics argues against infection as the primary driver (4, 15, 18, 19). The present case illustrates both the strength and the limits of the autoinflammatory model. The early biopsy was fully compatible with sterile chronic osteomyelitis yet offered no disease-specific diagnostic feature, so the diagnosis ultimately rested on the convergence of imaging, repeated tissue sampling, and the later emergence of characteristic cutaneous and axial disease. The response to IL-17A blockade, with improvement of an objective vertebral-corner lesion rather than only of symptoms, is consistent with a disease in which IL-17A is a dominant effector cytokine rather than an incidental participant.

The case also underscores that pathology in SAPHO syndrome can be nonspecific. Chronic inflammation, fibrosis, histiocytic infiltration, granulation tissue, and bone remodeling may be interpreted morphologically as chronic osteomyelitis. Such a description should not be equated automatically with active infection. In the correct clinical context, sterile osteomyelitis-like pathology can be part of SAPHO syndrome or chronic nonbacterial osteomyelitis. In this patient, the diagnostic shift required chronological integration of the anterior chest-wall pain, repeated sterile tissue findings, subsequent pustular disease, sacroiliitis, vertebral inflammatory lesions, and exclusion of key mimics.

The treatment course provides the main immunologic lesson. TNF inhibitors are used in SAPHO syndrome and may improve osteoarticular symptoms, but paradoxical psoriasiform or pustular eruptions have been reported during anti-TNF therapy (10). In this patient, pustular disease began soon after adalimumab was introduced and worsened with continued exposure. JAK inhibition subsequently improved the cutaneous eruption but did not prevent progression of axial osteitis. This dissociation between skin response and imaging activity was clinically important because it argued against judging disease control by skin findings alone.

The favorable response after IL-17A blockade is biologically plausible. The IL-23/IL-17 pathway participates in neutrophil-rich cutaneous inflammation, entheseal inflammation, and axial spondyloarthritis-spectrum disease, and IL-17A promotes osteoclast-mediated bone resorption through RANKL-dependent signaling, which provides a direct mechanistic bridge between the skin and bone domains of SAPHO syndrome. Although evidence in SAPHO syndrome remains limited to case reports, small series, and systematic reviews, IL-17A blockade may be particularly relevant when pustular skin disease coexists with sacroiliitis or vertebral-corner osteitis (5, 11, 19). Supporting experience is accumulating but remains heterogeneous: a four-patient series reported improvement in both articular and cutaneous domains, whereas a three-patient series found no appreciable osteoarticular benefit, and other reports describe partial skin responses only (11, 19). In this case, secukinumab was associated with improvement in MRI inflammation, gait, and skin control, supporting this mechanistic interpretation. Notably, the response occurred in a patient who had failed four sequential therapeutic strategies, including two agents with established efficacy in axial spondyloarthritis, which suggests that IL-17A blockade is not merely duplicating an existing mechanism but addressing a dominant cytokine in this particular phenotype. This interpretation is strengthened by the absence of pustular recurrence during maintenance, indicating that cutaneous and axial control were achieved concurrently rather than traded off against each other.

Treatment options in SAPHO syndrome remain heterogeneous, and no regimen has been validated in randomized controlled trials; treatment selection is therefore driven by which domain of disease predominates (5, 17–19). For pain-predominant disease, nonsteroidal anti-inflammatory drugs and analgesic therapy are first-line, and local measures, intra-articular corticosteroid injection, and physical therapy may be added; these measures are rarely sufficient alone. For bone-turnover-predominant disease with osteolysis and hyperostosis, bisphosphonates have the strongest supporting evidence, based on open-label experience with intravenous pamidronate in 10 and 22 patients, in which pain, disability, and marrow edema improved, particularly in spinal disease (12, 13). Corticosteroids are generally reserved for short bridging courses, because disease frequently flares during tapering, as occurred in this patient. Conventional disease-modifying antirheumatic drugs, including methotrexate, sulfasalazine, cyclosporine, and apremilast, have modest and inconsistent evidence; in the 164-patient cohort, 61% of patients received conventional disease-modifying drugs and 33% oral bisphosphonates, yet no standardized treatment algorithm emerged (16). Our own experience with apremilast and cyclosporine was consistent with that literature. Antibiotics directed at Cutibacterium acnes, such as doxycycline and minocycline, are reasonable when acne or pustulosis is prominent, but response rates are low and the antimicrobial contribution to SAPHO pathogenesis remains unproven (17, 19). Denosumab, which we administered for presumed tumor-like osteolysis, has only isolated case-level support in this setting and should not be interpreted as disease-targeted SAPHO therapy (19). Biologic and targeted agents beyond TNF blockade are increasingly used. TNF inhibitors remain the most extensively reported class, with osteoarticular responses in the majority of published cases, although paradoxical pustular and psoriasiform eruptions complicate their use, as in our patient (10, 19). Emerging alternatives with supportive case-level or small-series evidence include IL-1 blockade with anakinra, IL-6 receptor blockade with tocilizumab, IL-12/23 blockade with ustekinumab, IL-23p19 blockade with risankizumab, IL-17 blockade with secukinumab, ixekizumab, brodalumab, or bimekizumab, and JAK inhibition with tofacitinib, baricitinib, or upadacitinib (19). Ancillary approaches reported in small series include colchicine, topical and systemic anti-acne therapy, granulocyte-monocyte apheresis, and, in selected cases, surgical debridement or curettage of destructive lesions (18, 19). This observation does not imply that IL-17A blockade should replace established first-line approaches in all patients with SAPHO syndrome; rather, the practical implication from this case is more specific. When a patient has active axial osteitis together with pustular inflammation, particularly after paradoxical cutaneous worsening during TNF inhibition or a discordant skin-versus-bone response to JAK inhibition, IL-17A blockade deserves early consideration, and treatment decisions should be guided by objective imaging rather than by skin findings alone.

The limitations of this report should be acknowledged. This is a single-patient observation, and therapeutic response cannot prove mechanism or establish generalizable efficacy. The diagnosis was clinicopathologic rather than based on a disease-specific biomarker, and no validated diagnostic or classification criteria for SAPHO syndrome exist to anchor it independently; the modified Kahn criteria were used, and the differential with chronic nonbacterial osteitis and chronic recurrent multifocal osteomyelitis, with which SAPHO syndrome overlaps, cannot be resolved on the basis of this case alone (2, 4, 18). The comparative literature discussed above is itself limited to small series, case reports, and narrative syntheses, so the interpretation offered here is provisional and may change as larger registries and prospective cohorts mature (5, 17, 19). The patient-authored perspective is not yet available, and some early investigations were performed at outside hospitals. Nevertheless, the case advances the field by showing how SAPHO syndrome can enter the differential diagnosis of destructive FDG-avid osteolysis and how longitudinal treatment response, assessed with serial MRI, can help identify a dominant IL-17A-responsive inflammatory phenotype.

A further limitation is that the acetabular lesion was exposed to multiple interventions, including denosumab, antimicrobial therapy, surgery, and later immunomodulation, so improvement or progression at different sites cannot be attributed to one intervention with certainty. However, the key outcome emphasized here is not resolution of the original acetabular defect alone, but the later improvement of an active T12 inflammatory lesion after secukinumab in parallel with durable skin control. This temporal association is hypothesis-generating and should be interpreted in the context of future prospective data or registry-based studies.

## Conclusion

In patients with FDG-avid osteolytic bone destruction, SAPHO syndrome should be considered after malignancy, infection, and histiocytic disease are systematically excluded. Repeated sterile inflammatory pathology, antimicrobial failure, anterior chest-wall pain, pustular eruption, sacroiliitis, and vertebral-corner lesions supported the diagnosis in this patient. The clinical and MRI response to secukinumab suggests that IL-17A blockade may be a rational therapeutic strategy when SAPHO-related axial osteitis remains active, particularly in the setting of pustular inflammation and inadequate response or paradoxical worsening with other immunomodulatory approaches.

## Data Availability

All relevant clinical information is included in this article and the accompanying figures.
