# Supplementary material for: Case Report: Interleukin-17A blockade for SAPHO syndrome presenting as an FDG-avid destructive osteolytic acetabular lesion
Source: Front Immunol. 2026 Oct 23;17:1988760. doi: 10.3389/fimmu.2026.1988760 (PMC13739816; doi:10.3389/fimmu.2026.1988760)
Supplement: Supplementary file 1 [file Table1.docx]

**Supplementary Material**

Case Report: Interleukin-17A blockade for SAPHO syndrome presenting as an FDG-avid destructive osteolytic acetabular lesion

# Supplementary Figure Legends

Supplementary Figure 1. Serial MRI changes during treatment. (A) After 4 months of denosumab, MRI showed progression of abnormal signal in the left iliac wing, acetabulum, superior pubic ramus, and surrounding soft tissues. (B) Three months after discharge, after adalimumab discontinuation and treatment with doxycycline, methylprednisolone, and topical fusidic acid, MRI showed partial improvement. (C) During glucocorticoid tapering, after ineffective apremilast and cyclosporine, MRI showed recurrent progression of marrow and soft-tissue edema. (D) Six months after discharge, despite partial clinical response to upadacitinib, pelvic MRI showed progression to bilateral sacroiliitis, more prominent on the left. (E) Spine MRI showed inflammatory lesions at multiple vertebral-body corners, particularly T12, prompting escalation to secukinumab.

Supplementary Figure 2. Skin-biopsy findings. Hematoxylin and eosin staining of skin-biopsy specimens from pustular lesions showed epidermal hyperplasia and acanthosis, with lymphocytic infiltration around dermal vessels and hair follicles. Focal follicular dilatation with perifollicular histiocytic and multinucleated giant-cell deposition was present, findings consistent with granulomatous folliculitis in the context of SAPHO syndrome.

# Supplementary Table S1. Completed CARE Checklist

The checklist was completed against the latest manuscript draft. Because the draft does not contain journal line numbers, locations are reported by manuscript section and display item.

| **Topic** | **Item** | **CARE checklist item** | **Reported in latest draft** |
| --- | --- | --- | --- |
| Title | 1 | The diagnosis or intervention of primary focus followed by the words 'case report'. | Title page: 'Case Report: Interleukin-17A blockade for SAPHO syndrome presenting as an FDG-avid destructive osteolytic acetabular lesion'. |
| Keywords | 2 | Two to five key words that identify diagnoses or interventions, including 'case report'. | Keywords section: includes SAPHO syndrome, autoinflammation, sterile osteitis, osteolytic lesion, secukinumab, interleukin-17A, paradoxical pustulosis, and case report. Consider reducing to 5-6 if the target journal limits keywords. |
| Abstract | 3a | Introduction: what is unique and what does it add to the medical literature? | Abstract: states the atypical destructive osteolytic presentation and therapeutic lesson around IL-17A blockade. |
| Abstract | 3b | Main symptoms and important clinical findings. | Abstract and Case description: left hip pain, anterior chest-wall pain, destructive acetabular/iliac lesion, sterile osteitis-like pathology, pustular eruption, sacroiliitis, and T12 vertebral-corner lesion. |
| Abstract | 3c | Main diagnoses, therapeutic interventions, and outcomes. | Abstract: SAPHO syndrome; antimicrobial therapy, denosumab exposure, surgery, adalimumab, upadacitinib, and secukinumab; improvement after secukinumab. |
| Abstract | 3d | Conclusion: main take-away lessons. | Abstract and Conclusion: longitudinal clinicopathological reassessment and IL-17A blockade as a rational option when axial osteitis and pustular disease coexist. |
| Introduction | 4 | Brief background summarizing why this case is unique, with medical literature references. | Introduction: describes SAPHO syndrome, overlap with sterile osteitis/chronic nonbacterial osteomyelitis, and diagnostic challenge of FDG-avid osteolytic lesions with references. |
| Patient information | 5a | De-identified demographic and patient-specific information. | Case description: 27-year-old woman; de-identified clinical presentation. |
| Patient information | 5b | Primary concerns and symptoms of the patient. | Case description: 7-month left hip pain and intermittent left anterior chest-wall pain. |
| Patient information | 5c | Medical, family, and psychosocial history, including relevant genetic information. | Case description: relevant negative symptoms are included. Ensure the submitted draft retains past history, family history, occupation/social history, and allergy details if required by Frontiers. |
| Patient information | 5d | Relevant past interventions and their outcomes. | Case description and Table 1: prior NSAID/topical analgesic therapy, denosumab, antimicrobial therapy, surgery, adalimumab, apremilast, cyclosporine, upadacitinib, and secukinumab with outcomes. |
| Clinical findings | 6 | Relevant physical examination and other clinical findings. | Case description: clinical presentation and relevant systemic negatives. Add vital signs and focused hip examination if not retained in the submitted version. |
| Timeline | 7 | Important dates and times in the case. | Table 1 in manuscript and Supplementary Table S1. |
| Diagnostic assessment | 8a | Diagnostic methods, including physical examination, laboratory testing, imaging, and pathology. | Case description, Diagnostic assessment, Figures 1-2, Table 1, and Supplementary Table S1. |
| Diagnostic assessment | 8b | Diagnostic challenges, such as access, financial, or cultural issues. | Diagnostic assessment: destructive FDG-avid lesion, nonspecific chronic osteomyelitis-like pathology, and low-confidence mNGS signal with possible contamination. |
| Diagnostic assessment | 8c | Diagnostic reasoning, including other diagnoses considered. | Diagnostic assessment: carcinoma/metastasis, lymphoma, Langerhans-cell histiocytosis, histiocytic disorders, bacterial/mycobacterial/fungal infection, and SAPHO syndrome. |
| Diagnostic assessment | 8d | Prognostic characteristics when applicable. | Diagnostic assessment and follow-up: no disease-specific prognostic biomarker; objective MRI activity and treatment response were used for monitoring. |
| Therapeutic intervention | 9a | Types of intervention, including pharmacologic, surgical, preventive, and self-care interventions. | Therapeutic intervention, follow-up, and outcomes; Table 1; Supplementary Table S1. |
| Therapeutic intervention | 9b | Administration of intervention, including dosage, strength, and duration. | Therapeutic intervention and Table 1: denosumab 120 mg every 4 weeks; adalimumab 40 mg every 2 weeks; upadacitinib 15 mg daily; secukinumab 150 mg weekly for five doses, then every 2 weeks, then every 3 weeks. Antimicrobial agents are listed; add exact intravenous doses if available. |
| Therapeutic intervention | 9c | Changes in intervention and rationale. | Therapeutic intervention: antimicrobial failure, adalimumab-associated pustular eruption, inadequate control with apremilast/cyclosporine/upadacitinib, escalation to secukinumab for active axial osteitis. |
| Follow-up and outcomes | 10a | Clinician- and patient-assessed outcomes. | Therapeutic intervention and Conclusion: rash control, improved gait, improved T12 inflammatory lesion, stable disease during secukinumab maintenance. |
| Follow-up and outcomes | 10b | Important follow-up diagnostic and other test results. | Therapeutic intervention, Figure 3, Table 1, and Supplementary Figure 1: serial pelvic and spine MRI changes; ESR elevation during active disease. |
| Follow-up and outcomes | 10c | Intervention adherence and tolerability. | Therapeutic intervention: secukinumab continued with interval extension to every 3 weeks; no recurrent rash reported. |
| Follow-up and outcomes | 10d | Adverse and unanticipated events. | Therapeutic intervention and Figure 3: pustular eruption after adalimumab initiation, progressive with continued exposure. |
| Discussion | 11a | Strengths and limitations of the management of this case. | Discussion: repeated tissue sampling, longitudinal reassessment, single-patient limitation, lack of disease-specific biomarker, outside-hospital testing, and multiple sequential interventions. |
| Discussion | 11b | Relevant medical literature. | Discussion and References: SAPHO syndrome, chronic nonbacterial osteitis, anti-TNF paradoxical pustulosis, IL-17/IL-23 targeted therapy, bisphosphonate therapy, and infection/histiocytosis differentials. |
| Discussion | 11c | Scientific rationale for conclusions, including assessment of possible causes. | Discussion: sterile osteitis-like pathology, evolving osteoarticular and cutaneous phenotype, axial inflammation despite JAK inhibition, and response to IL-17A blockade. |
| Discussion | 11d | Primary take-away lessons. | Conclusion and final Discussion paragraphs: IL-17A blockade may be rational when SAPHO-related axial osteitis and pustular inflammation coexist, after malignancy and infection are excluded. |
| Patient perspective | 12 | Patient perspective or experience, when appropriate. | Patient perspective section: written informed consent is reported; patient-authored narrative perspective is not yet available and should be inserted if obtained before submission. |
| Informed consent | 13 | Did the patient provide informed consent? | Ethics statement and Patient perspective: written informed consent was obtained for publication of the case and deidentified clinical images. |
